# Comparative Antitumor Effect of Preventive *versus* Therapeutic Vaccines Employing B16 Melanoma Cells Genetically Modified to Express GM-CSF and B7.2 in a Murine Model

**DOI:** 10.3390/toxins4111058

**Published:** 2012-10-31

**Authors:** Antonio Miguel, María José Herrero, Luis Sendra, Rafael Botella, Rosa Algás, Maria Sánchez, Salvador F. Aliño

**Affiliations:** 1 Department of Pharmacology, Faculty of Medicine, University of Valencia, Avda. Blasco Ibáñez, 15, Valencia 46010, Spain; Email: matasantonio@hotmail.com (A.M.); luissendra@hotmail.es (L.S.); sdelvall@post.uv.es (M.S.); 2 Instituto Investigación Sanitaria La Fe, Bulevar Sur s/n, Valencia 46026, Spain; 3 Dermatology, Hospital Universitario y Politécnico La Fe, Bulevar Sur s/n, Valencia 46026, Spain; Email: rbotellaes@gmail.com; 4 Radiotherapy, Hospital Clínico Universitario, Av. Blasco Ibáñez, 10, Valencia 46010, Spain; Email: algas_amp@gva.es; 5 Clinical Pharmacology Unit, Hospital Universitario y Politécnico La Fe, Bulevar Sur s/n, Valencia 46026, Spain

**Keywords:** cancer vaccines, gene therapy, non-viral, GM-CSF, B7.2

## Abstract

Cancer vaccines have always been a subject of gene therapy research. One of the most successful approaches has been working with genetically modified tumor cells. In this study, we describe our approach to achieving an immune response against a murine melanoma model, employing B16 tumor cells expressing GM-CSF and B7.2. Wild B16 cells were injected in C57BL6 mice to cause the tumor. Irradiated B16 cells transfected with GM-CSF, B7.2, or both, were processed as a preventive and therapeutic vaccination. Tumor volumes were measured and survival curves were obtained. Blood samples were taken from mice, and IgGs of each treatment group were also measured. The regulatory T cells (Treg) of selected groups were quantified using counts of images taken by confocal microscopy. Results: one hundred percent survival was achieved by preventive vaccination with the group of cells transfected with p2F_GM-CSF. Therapeutic vaccination achieved initial inhibition of tumor growth but did not secure overall survival of the animals. Classical Treg cells did not vary among the different groups in this therapeutic vaccination model.

## 1. Introduction

There is increasing evidence of the importance of immunotherapy in the fight against cancer. Currently, certain cytokines are being used as adjuvants in the treatment of some types of cancer. Melanoma is possibly the best candidate for immunotherapy as an alternative to current treatments because it is a very immunogenic type of malignancy.

In the field of immunotherapy, antitumor vaccines are one of the most promising therapeutic strategies. Antitumor vaccines with genetically modified cells have already proved to be effective in application to some types of cancer in preclinical models, and some of them are being tested in clinical trials [1,2,3,4,5,6,7,8,9,10,11,12,13,14,15,16,17,18,19,20,21,22,23,24]. A number of cytokines have already shown a clear antitumor effect. The cytokine GM-CSF (granulocyte and macrophage colony stimulating factor) has demonstrated a very important antitumor effect [14,15,16,17,18,19,20,21,22,23,24,25,26,27,28]. GM-CSF is produced by a wide range of cell types, and its main functions are to stimulate the proliferation, maturation, and function of APCs (Antigen Presenting Cells) [29], facilitating their presentation of antigen to T cells, and contributing in this way to the immune cellular response against tumors.

However, the signaling pathways of the immune system are very complex, and, in the last few years, many studies have tested different combinations of cytokines with other molecules in order to improve antigen presentation, such as the membrane surface costimulatory molecule B7.2 [30,31,32,33,34,35,36]. This molecule, also known as CD86, is expressed on the surface of APCs and is necessary for antigen recognition in the context of major histocompatibility antigen for the activation of T lymphocytes. It has demonstrated its utility in some models of antitumor vaccines, alone [35,36] or in combination with GM-CSF [33,34], as we also prove in the present work.

Previous studies in our laboratory have shown the importance of the order of antigen and cytokine presentation in a model of preventive vaccination against murine melanoma—the best results being obtained when featuring tumor antigens first, and then the cytokine, or both simultaneously [27]. In addition, the vaccination was shown to be most effective when the cytokine was produced by the same genetically modified tumor cell rather than administered independently [37]. We have previously achieved total survival of B16 melanoma-bearing mice in a preventive vaccine model using freshly GM-CSF transfected B16 cells [25,27,38]. Here, we aimed to take advantage of the fact that the B7.2 molecule has also already demonstrated its usefulness in some antitumor vaccine models, and that its nature as a surface molecule could make it useful for manipulating and selecting the effectively transfected cells in future experiments. We thus transfected B16 cells with a bicistronic plasmid, containing both *mGM-CSF* and *mB7.2* genes. Accordingly, we expected that the transfected cells, expressing B7.2 on their surface, would also express GM-CSF; B7.2 expression therefore could be very useful when it comes to manipulating and/or characterizing cells. 

The present study describes the efficacy of the GM-CSF transfected cells vaccine, and the effect of this cytokine in combination with the costimulator molecule B7.2, with a view to determining whether there is some kind of synergy between them. This has been done by assessing efficacy in an antitumor cell preventive vaccine, though also assuming the challenge of a therapeutic vaccination. The importance of the amount of antigen and cytokine in the antitumor response has also been evaluated, employing vaccines with different doses of tumor transfected cells. Finally, a study has been made of the presence of classical regulatory T cells (Treg) in the setting of the therapeutic vaccine, in order to try to clarify whether these cells are responsible for the failure of the antitumor immune response once the tumor has become established.

## 2. Results and Discussion

### 2.1. Preventive Vaccination

**Figure 1:**
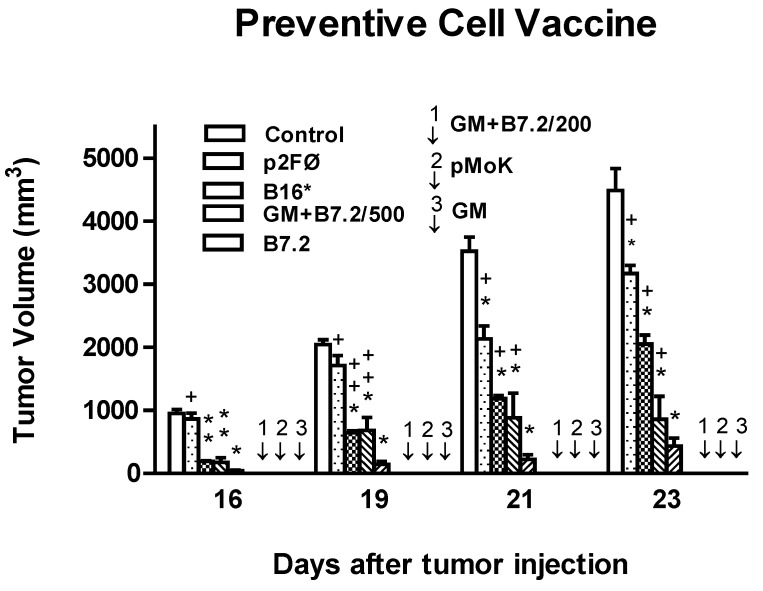
Tumor volume in preventive vaccination. Results from inhibition of tumor volume with vaccination groups: (**a**) Control; (**b**) B16-p2fØ; (**c**) B16*; (**d**) B16-GM-CSF + B7.2/500; (**e**) B16-B7.2; (**f**) B16-GM-CSF + B7.2/200; (**g**) B16-pMok_GM-CSF; (**h**) B16-GM-CSF. Mice were injected with 10^5^ B16 wild cells in the left leg. We used a vaccination dose of 2 × 10^5^ cells, but also tested other doses in the treatments with B16-GM-CSF + B7.2, expressing the number of cells used with 200 or 500, corresponding to 2 × 10^5^ or 5 × 10^5^ cells, respectively. In the figure, “*” corresponds to the maximum statistical difference, *p* < 0.001, and “**” to *p* < 0.01, both with respect to the control group. In turn, “+” corresponds to the maximum statistical difference, *p* < 0.001, and “++” to *p* < 0.01, both with respect to the B16-GM-CSF group. Arrows identified as 1, 2 and 3 represent groups B16-GM-CSF + B7.2/200, B16-pMok_GM-CSF and B16-GM-CSF, respectively, with total inhibition of tumor growth during the measurement period.

The tumor volume in each treatment group is represented in Figure 1. The best results were obtained with groups B16-GM-CSF, B16-pMok_GM-CSF, and B16-GM-CSF + B7.2/200 (marked with arrows in the figure), where there was no visible development of the tumor implanted during the measurement period—a time in which mice from other groups had already begun to die as a result of tumor development. It should be noted that these results were reached in these three groups vaccinated with 2 × 10^5^ cells, underscoring that the B16-GM-CSF + B7.2/200 group was producing less than half the amount of GM-CSF produced by the other two groups (data not shown), as transfection was simultaneous with two genes and so the production of each gene was reduced as compared with transfections for only one gene (data not shown). 

**Figure 2:**
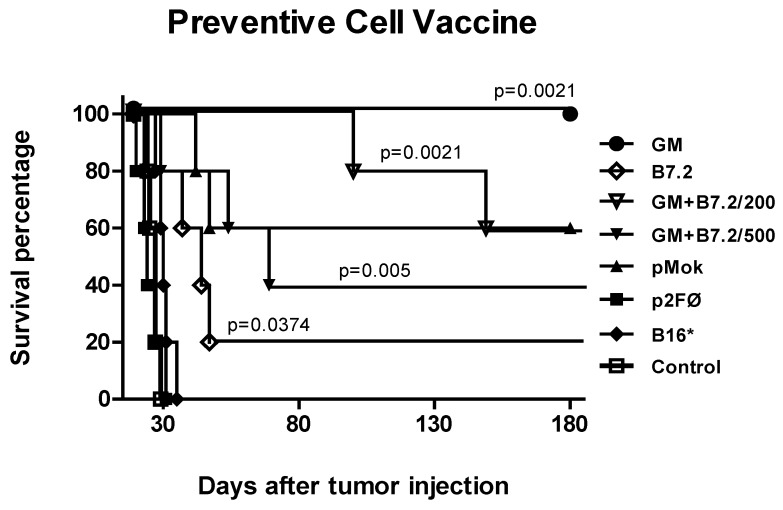
Survival in preventive vaccination. The plot shows survival of the groups described in Figure 1.

Survival curves are shown in Figure 2. In the majority of cases, the curves were consistent with the inhibition of tumor growth, whereby the groups with smaller tumor sizes survived longer. Such survival was particularly notorious in two of the groups in which the tumor was not initially detectable. Only the B16-GM-CSF group maintained 100% survival of the animals more than six months after introduction of the tumor. In contrast, percentage survival in the B16-GM-CSF + B7.2/200 group was 80%. The B16-pMok_GM-CSF group did not reach a better result than the other groups which already showed tumor growth in Figure 1 (60% survival). For this reason, we decided to perform the following experiments regardless of plasmid pMok_mGM-CSF, since it did not afford any advantages over p2F-mGMCSF, which showed the best performance in our vaccine model. The other groups reached survival rates of between 20% and 60%, while B16* and p2FØ were not differentiated from the control group. 

**Figure 3:**
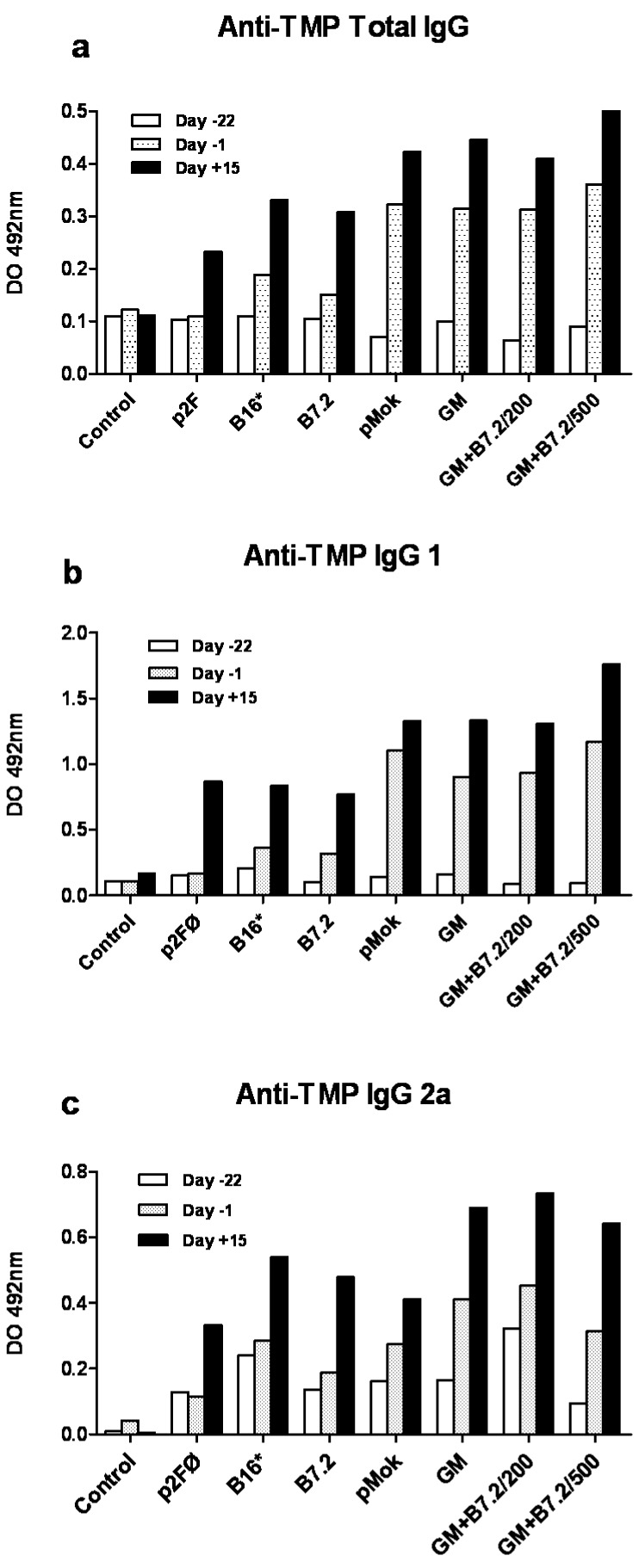
Production of total IgG (a) and IgG1 (b) and IgG2a (c) subtypes against TMP in preventive vaccination. After blood samples were taken from the animals on day −22, day −1 and day 15, with respect to the day of implantation of the tumor (day 0), and the plasma was obtained, the latter was used for enzyme linked immunosorbent assaying (ELISA) specific total IgG (a) and subtypes IgG1 (b) and IgG2a (c) against TMP. The samples were assayed in duplicate to calculate the mean and standard deviation, which is too small to see in the figure. The treatment groups are described in Figure 1. All groups showed significant differences *versus* the control as a minimum at day −1 and day 15 (*p* < 0.001).

The results referred to total specific anti-TMP IgG are reflected in Figure 3a, where groups that produced GM-CSF reached a higher production of IgG and usually with a greater difference between day −1 and day 15 of production; thus, it appears that GM-CSF induces earlier stimulation than B7.2.

The IgG subtypes, as reflected in Figure 3a, b, also offered the same overall results as total IgG, especially in the case of IgG2a. Transfection with GM-CSF contributed to the effectiveness of the antitumor response in a meaningful way, while isolated B7.2 produced more limited effects. The combination of B7.2 with GM-CSF did not exceed the effect achieved with GM-CSF alone, so evidently the positive effects upon total efficacy of the combination do not lie in IgG production. 

Tumor Reimplantation: Verification of Immunological Memory 

One year after the beginning of the previous experiment, we rechallenged the surviving mice once again with the same tumor, to determine whether the immune response that had been established and allowed them to survive had generated enough immunological memory to protect them from a second exposure to the tumor.

**Figure 4:**
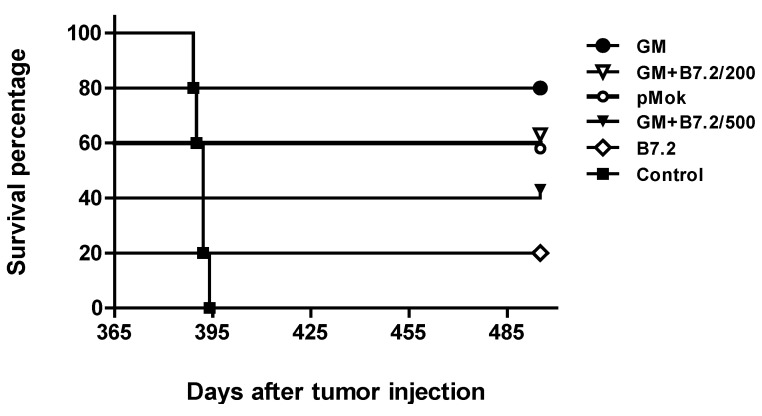
B16 tumor reimplantation. One year after first vaccination with transfected cells, a reminder vaccine dose was administered to the surviving animals, which were again introduced to the tumor (day 0 = day 365, 10^5^ B16 wild type cells). The plot shows the survival of these animals and a mice control group not previously treated.

The experiment began with only one vaccine dose with transfected cells in the remaining three mice in the B16-pMok_GM-CSF group, three mice in the B16-GM-CSF + B7.2/200 group, two mice in the B16-GM-CSF + B7.2/500 group, a single mouse in the B16-B7.2 group, and four mice in the B16-GM-CSF group. During the time from the end of the previous experiment to the start of this new experiment, some animals died due to causes unrelated to the first implanted tumor, as confirmed by the necropsy study. All the mice were free of tumor at the time of the beginning of this new experiment. We employed new animals as control group (*n* = 5), with no previous treatment, and of similar age to the age of the survivors of the previous experiment (approximately 14 months). Tumor cells (10^5^ B16 cells, in the left leg) were administered 14 days after the vaccine. The survival results are shown in Figure 4.

**Figure 5:**
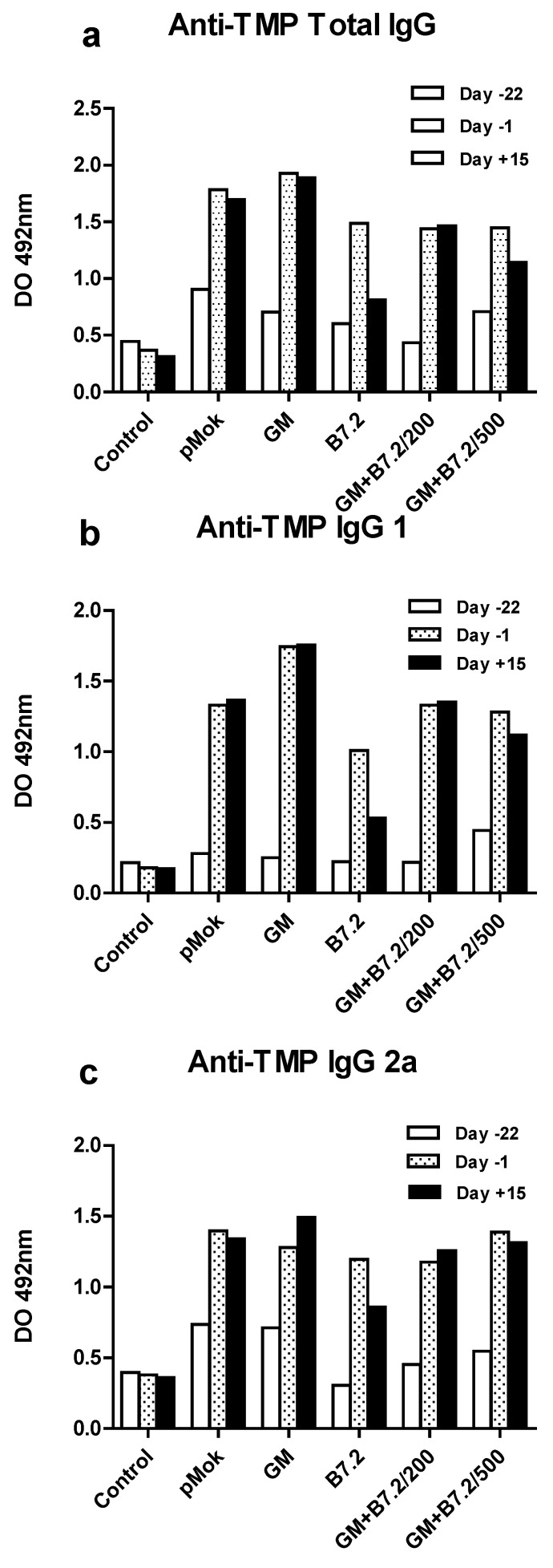
Production of total IgG (a) and IgG1 (b) and IgG2a (c) subtypes specific to TMP in reimplantation with preventive vaccination. The mice surviving the first cell vaccine received a vaccine reminder dose and the introduction of tumor 14 days later. Blood was extracted according to the usual pattern (days −22, day −1 and day 15) with respect to implementation of the tumor, day 0. Retrieved plasma was used in the ELISA test to detect IgG anti-TMP, as in Figure 3. The plot shows the production groups vaccinated with B16 cells transfected with p2F_m-GM-CSF, p2F_mGM-CSF + mB7.2 dose 2 × 10^5^ and 5 × 10^5^, p2F_m-B7.2, and the control group.

All the treated mice survived second tumor implantation. All of them died of old age—the necropsy study confirming that death had not been due to B16 tumor. The three usual blood samples were also taken in this experiment to evaluate total IgG production and the production of subtypes IgG1 and IgG2a. The results are shown in Figure 5a, b, c, respectively. The immune response of the surviving animals was faster and more powerful after second exposure to the tumor, which demonstrates an effective immunological memory response against the tumor.

### 2.2. Therapeutic Vaccination

#### 2.2.1. Therapeutic Antitumor Efficacy of Cellular Therapy at Low Doses

We evaluated two different doses of transfected cells, 5 × 10^5^ or 2 × 10^6^ per dose, per mouse. The vaccination groups were: (a) control (the same volume of only DMEM as that employed to resuspend cells in each dose); (b) B16-p2FØ, 2 × 10^6^; (c) B16-GM-CSF, 5 × 10^5^; (d) B16-GM-CSF, 2 × 10^6^; (e) B16-GM-CSF + B7.2, 5 × 10^5^; and (f) B16-GM-CSF + B7.2, 2 × 10^6^.

**Figure 6:**
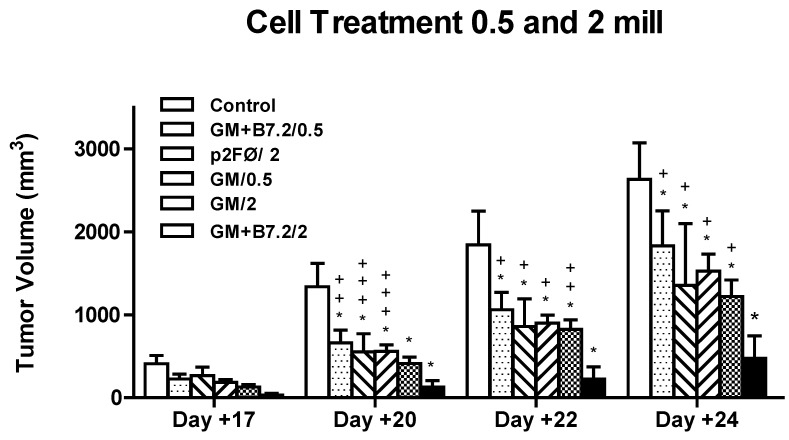
Inhibition of tumor growth in cell treatment at low doses. C57BL/6 mice (*n* = 5 per group) were vaccinated after tumor implantation (day 0, 2 × 10^4^ B16 wild type cells) 3, 10 and 17 days. Treatment groups were control (only DMEM) or 5 × 10^5^ or 2 × 10^6^ cells transfected with plasmid p2F (Ø, GM-CSF, GM-CSF + B7.2). The tumor’s size was measured. The symbol * represents a statistically significant difference (*p* < 0.001) with respect to the control group. In turn, “+” corresponds to the maximum statistical difference, *p* < 0.001, “++” to *p* < 0.01, and “+++” to *p* < 0.05, with respect to the B16-GM-CSF + B7.2/2 group.

As seen in Figure 6, all treatment groups reached a difference with the highest degree of significance *versus* the control group, but the best treatments were GM-CSF alone and especially combined with the B7.2 molecule, with the dose of 2 × 10^6^ cells. The GM+B7.2/2 group showed tumor growth inhibition equal to or greater than 80% with respect to the control group. In addition, GM + B7.2/2 significantly differed in the two last days with GM/2 (*p* < 0.001).

**Figure 7:**
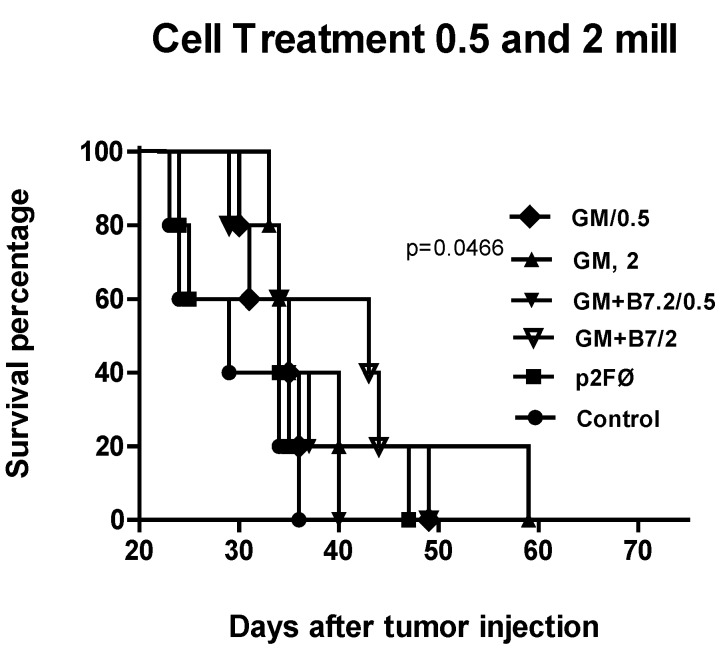
Survival in cell treatment at low doses. Mortality among mice treated according to the groups listed in Figure 6 is shown in the figure.

The survival results are shown in Figure 7. None of the animals reached overall survival, but the results in the treated groups were always better than those in the control group. The groups treated with 5 × 10^5^ cells were not very different among each other, except for the last mouse in the GM/0.5 group, which increased final survival by approximately 15 days *versus* the control group. The three groups treated with 2 × 10^6^ cells also showed quite similar behavior in terms of survival, not reflecting the differences between groups that were observed in tumor volume inhibition. However, GM/2 did not reach such significance, though the behavior of the group was very similar.

**Figure 8:**
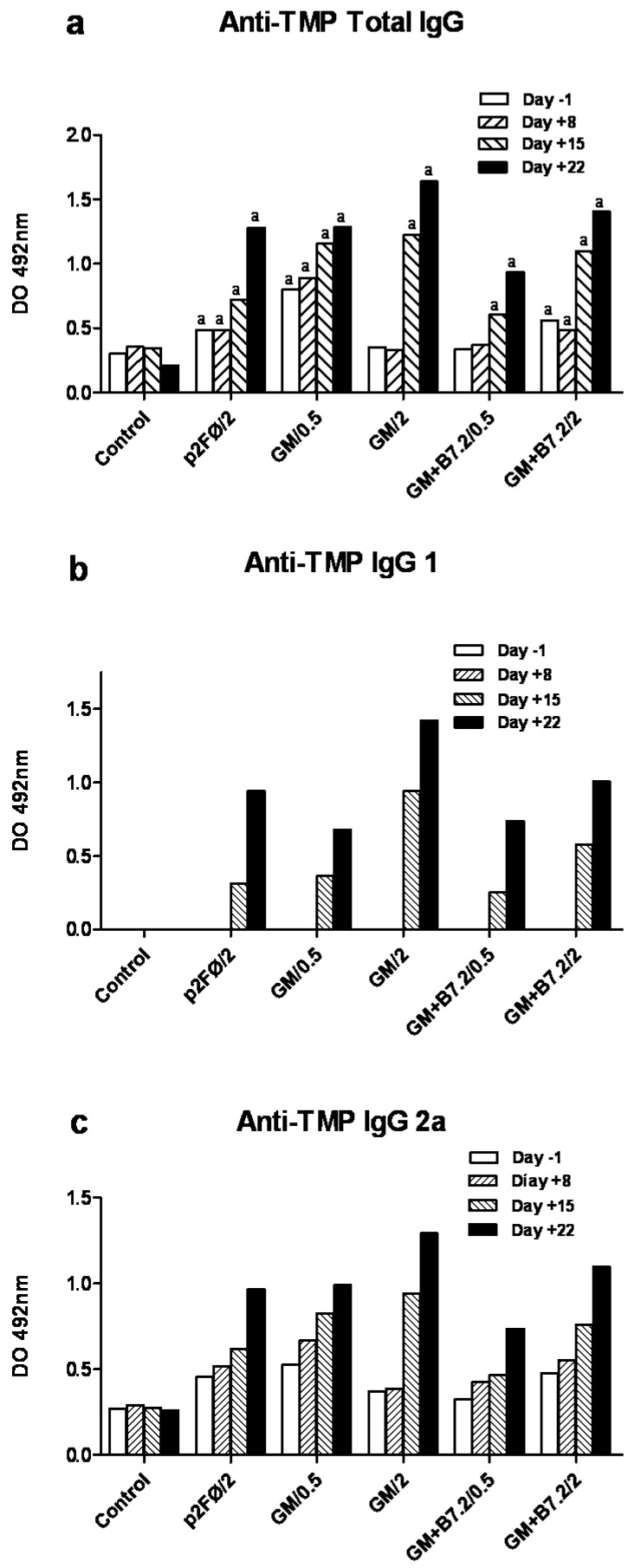
Production of specific total IgG (a) and IgG1 (b) and IgG2a (c) subtypes against TMP in low dose cell treatment. After implanting tumor in animals on day 0 with 2 × 10^4^ B16 cells, three vaccination doses were administered on days 3, 10 and 17, and four blood extractions were performed on days 3, −1, 8, 15 and 22. With the retrieved plasma, ELISA testing was made of samples in duplicate, calculating the mean and standard deviation, which is too small to see in the figure. “*” represents *p* < 0.001 *versus* the control group. Groups described in Figure 6.

Figure 8 shows the production of specific IgG against TMP, with total IgG in Figure 8a, IgG1 in Figure 8b, and IgG2a in Figure 8c. In relation to total IgG, the two groups that yielded the best survival results, having been treated with 2 × 10^6^ cells (GM-CSF and GM-CSF + B7.2), also showed greater immunoglobulin production, which was particularly increased in the last two blood samples, collected on days +15 and 22.

#### 2.2.2. Therapeutic Antitumor Efficacy at High Cell Dose

Based on the results of previous treatments, we decided to continue increasing the dose of cells. Our treatment groups this time comprised 8 × 10^6^ cells per dose, per mouse. The vaccination groups were: (a) control (the same as in previous vaccinations); (b) B16*, B16 irradiated cells; (c) B16-p2FØ; (d) B16-B7.2; (e) B16-GM-CSF; and (f) B16-GM-CSF + B7.2.

**Figure 9:**
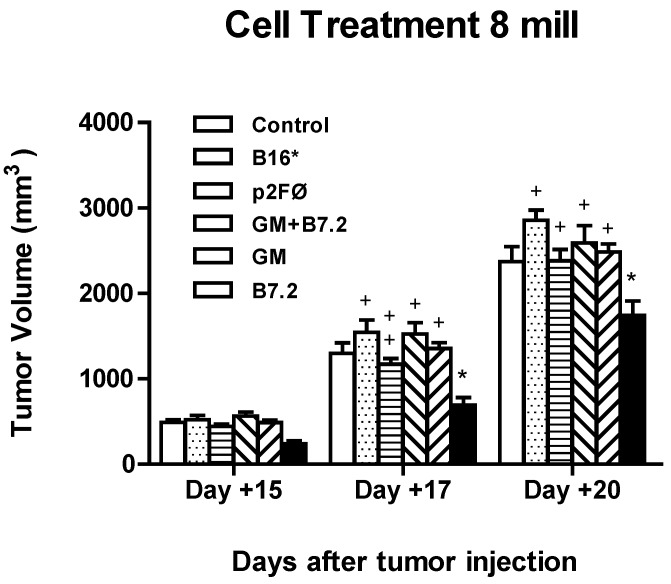
Inhibition of tumor growth in high dose cell treatment. Mice (*n* = 5 per group) were vaccinated after tumor implantation (day 0, 2 × 10^4^ B16 cells) day 3, 10 and 17 with 8 × 10^6^ transfected cells, per dose (only DMEM in the control group or irradiated B16 without transfection in B16*), transfecting with plasmids p2FØ, p2F_mGM-CSF + mB7.2, p2F_mGM-CSF, and p2F_mB7.2, respectively. Tumor size was measured and statistical significance was calculated as in the rest of experiments. The symbol “*” represents statistical difference (*p* < 0.001) with respect to the control group. In turn, “+” corresponds to the maximum statistical difference, *p* <0.001, and “++” to *p* < 0.01, both with respect to the B16-B7.2 group.

As shown in Figure 9, the maximum reduction in tumor volume achieved with 8 × 10^6^ cells did not exceed 25% *versus* the control group, compared with almost 80% in the vaccination with 2 × 10^6^ cells transfected with p2F_mGM-CSF + mB7.2. In this experiment, the group that achieved statistical significance with respect to the control group (*p* < 0.001) had been treated with B7.2-producing cells, while the groups treated with GM-CSF-producing cells or their combination with B7.2 did not differ from the control group. 

These differences in tumor growth inhibition between the groups were not sufficient to cause any differences in survival outcomes (results not shown), as evidenced by the Kaplan-Meier and log-rank tests.

While treatments with 5 × 10^5^ and 2 × 10^6^ transfected cells achieved approximately 12 and more than 30 days, respectively, of delayed mortality *versus* the control group, when the cell dose was increased to 8 × 10^6^, a maximum delay of five days was obtained (GM-CSF), which was not sufficient to reach a statistically significant difference with respect to the control group.

The confocal microscopy study of regulatory T cells in the peripheral blood of treated animals, following counts of various fields (from 5 to 10 fields/treatment group) chosen at random, was able to identify CD4^+^ and CD8^+^ T cells and “classical” Treg cells (CD4^+^CD25^+^Foxp3^+^), but also identified an additional subpopulation showing CD4^−^CD25^+^Foxp3^+^ staining. 

There were no significant differences in the percentage of classical Treg cells among the different groups (data not shown). The cell type that actually showed most differences corresponded to the CD4^−^CD25^+^Foxp3^+^ group. On day 8, sample 1, these cells were almost imperceptible, becoming patent in samples 2 and 3, and reaching values even higher than in classical Treg with respect to the total number of cells, in the groups B16-B7.2, B16-GM-CSF and B16-GM-CSF + B7.2, where also statistical significance was obtained regarding percentage of cells as compared to Control group. 

**Figure 10:**
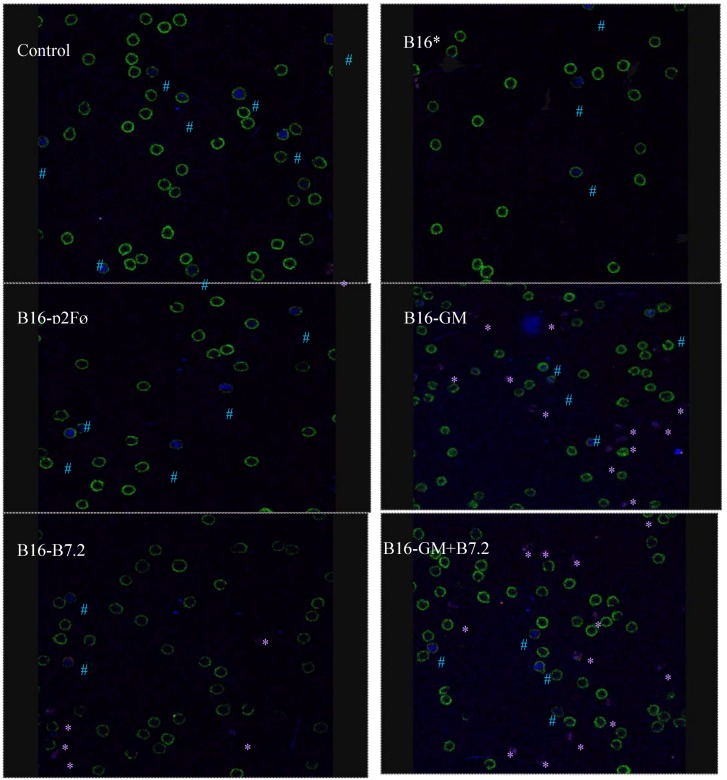
Representative confocal microscopy images in blood sample 3. Green CD4^+^, red CD25^+^, blue Foxp3^+^. The symbol # shows CD4^+^CD25^+^Foxp3^+^ cells, the symbol * shows CD4^−^CD25^+^Foxp3^+^ cells. Groups described in Figure 9.

The confocal microscopy images shown in Figure 10 illustrate the results of the different treatments in sample 3.

In this work we achieved total efficacy with preventive vaccination in the group with p2F-GMCSF transfected cells. 

Interestingly, and in accordance with published data, the group with pMoK_mGM-CSF transfected cells, whose net production of GM-CSF was higher than in cells transfected with p2F_mGM-CSF (data not shown), obtained comparatively poorer results. This difference was not initially evident in tumor growth inhibition, but was patent in the final survival results. While the B16-GM-CSF group retained 100% survival after more than six months of follow-up, the B16-pMok_GM-CSF group reached 60% survival at the end of the same period. Once again, these results support the idea of the counterproductive effect of an excess of GM-CSF [26,39].

On the other hand, GM-CSF in combination with B7.2 seemed to show a synergistic effect, since the B16-GM + B7.2/200 group, with the same number of cells as in the B16-GM-CSF group but with half the cytokine production, reached almost the same survival rate (80%). Doubling the cell dose in this treatment (B16-GM + B7.2/500 group) in order to achieve the same GM-CSF production as in the B16-GM-CSF group resulted in a poorer response, reducing survival to 40%, probably due to the excess of antigen and suppressor associated phenomena.

Production of specific immunoglobulins against the tumor was correlated to the survival results, this being most apparent in the case of IgG2a, where the more productive groups reached the best survival percentages: B16-GM-CSF and B16-GM-CSF + B7.2/200 showed the highest production values at day +15. 

A year after these experiments, the animals that had survived were rechallenged with the tumor, receiving only one vaccine dose. All vaccinated animals survived this second tumor introduction, remaining free of B16 tumor until natural death.

In all cases, IgG production was greater than in the control group, and the net production of IgG2a reached higher values at tumor reimplantation *versus* the first levels, one year earlier. In addition, the maximum values for both IgG1 and IgG2a were reached virtually in all groups at day −1 instead of day 15, indicating that the specific response was triggered earlier than on first exposure to the tumor. 

Vaccine-induced tumor immunity is known to be mediated primarily through the cellular immune compartment. T cell assays for B16 antigen-specific immunity would better speak to the immunogenicity of the vaccines (*i.e.*, ELISA, ELISPOT, tetramer staining). But our aim by measuring anti-B16 specific humoral immunity was different: these assays were incorporated as complementary information about the immunoglobulin switch as a cellular memory indicator. Our aim in the vaccination experiments has always been to achieve a model where we could perform the fullest possible monitoring of the entire process, getting as much information as possible. For the cytotoxicity studies we would have needed to sacrifice animals before their real endpoint and that would not have allowed us to fully track their antitumor response.

In these preventive vaccine experiments, and in addition to achieving maximum efficacy (total prevention of tumor development), two ideas were confirmed, supporting previous results of our own group and of other authors: (1) above a certain threshold, higher levels of GM-CSF production are not necessarily more effective, and may even prove counterproductive [26,27,39,40]; (2) there is some kind of synergistic effect between GM-CSF and B7.2 [30,31,32,33]. Two situations reflect this in our experiments, *i.e.*, the fact that the preventive model with B16-pMok-GM-CSF, producing more cytokine than B16-p2F-GM-CSF, did not work better, and the fact that B16 transfected with the bicistronic plasmid p2F-GM-CSF + B7.2 worked practically as well as p2F-GM-CSF alone. The promoter of GM-CSF gene in p2F plasmids is the ferritin promoter, which usually has no strong control. Its expression is constitutive, regular and sustained. In all experiments performed in our laboratory with p2F plasmids, we observed a decrease in GM-CSF production when this gene was accompanied by another in the same plasmid (bicistronic). We do not know the exact cause for the decrease in expression, but think that it might be due to steric impediments that prevent the transcription complex machinery from working more efficiently when there are two genes to be transcribed.

In the low cell dose treatment, we observed some kind of synergistic effect between GM-CSF and B7.2. However, this was lost in the high cell dose treatment. We think that this is due to the fact that when increasing the cell number, we are also increasing the quantity of secreted cytokine, and probably the quantity of GM-CSF secreted in this experiment proves excessive and results in a counterproductive effect. This is consistent with other publications [24,25,26,27,28,29,30,31,32,33,34,35,36,37,38,39], and we understand that it is also the reason why the treatment that works best in high cell dose vaccination is B16-B7.2, which does not express GM-CSF in itself, but possibly benefits from bystander (and not high) GM-CSF production in the tumor-vaccine milieu.

On the other hand, these results favor the useful role of transfecting the vaccine cells with *mB7.2* gene in order to take advantage of the surface position in the cells. As our group has also demonstrated [41], the expression of this molecule allows efficient selection and purification of transfected cells. By means of flow cytometry and/or magnetic beads-based systems, it is possible to identify and enrich the population of genuinely transfected cells, discarding the nontransfected cells after a classical transfection protocol.

Following success with the preventive vaccines, we developed a model closer to the real situation found in clinical practice: therapeutic vaccination. In this study, we recorded a reduction in tumor growth, but one which did not improve overall survival—though all groups showed maximum statistical differences with respect to the control group. The B16-GM + B7.2/2 group reached the highest tumor growth inhibition (up to 80% *versus* the control group). This group also showed a significant difference in the survival of animals, which was not recorded in any other case. 

These results and those of other groups suggest that perhaps the immune response elicited by the vaccine was being surpassed by some system inhibiting it. Many studies have been published in this regard in recent years [42,43,44,45], describing a group of new factors that may be involved in phenomena of this kind: regulatory T cells. B7.2 binds to CD28 and CTLA-4, and its binding to these receptors mediates distinct biologic functions [46,47]. Treg cells express CTLA-4 constitutively, and interestingly, as opposed to the widely demonstrated positive effect of CD28 ligation in T-cell activation and survival, it has also been reported that CTLA-4 costimulation delivers downregulatory signals, either by inhibiting signaling through the TCR or by inducing cell cycle arrest [48]—so this double role of B7.2 must also be further studied in our model. 

It is currently well supported that initial activation through recognition of antigen by T cells generates stimulatory signals when occurring in the presence of CD28/B7 costimulatory molecules. Binding of the CD28 ligand to the B7 molecule triggers a series of intracellular signals that lead to the formation of regulatory T cells (Treg) with immunosuppressive activity. These Treg cells produce a tolerance response to tumor cells, favoring their escape from the immune system and allowing tumor growth. Therefore, the depletion of these cells in the study of cancer has become a topic of great interest. In an attempt to increase the effectiveness of vaccines, avoiding the limitation created by tolerance, depletion of classical Treg cells has been chosen in many studies, either nonspecifically with cyclophosphamide, or selectively with specific monoclonal antibodies (anti-CD25 [49,50,51,52,53], anti GITR [54], anti-CTLA4 [55,56]). These strategies have also been moved to humans in other clinical settings, employing a specific compound for the selective depletion of human Treg, where drugs such as ONTAK (Dinileukin diftitox)—a conjugate of diphtheria toxin and IL-2 (CD25 is part of the IL-2 receptor)—are being used [57,58]. However, depletion attempts have not always achieved total depletion, and the final results against cancer are ambiguous [43,57,58]. Much effort therefore remains to be made in this area.

Currently, other costimulatory molecules such as 4-1BBL [59,60,61] and OX40L [62] are being tested to evaluate their usefulness in cancer treatment. These molecules have demonstrated their ability to increase activation of naive T cells and acquire effector function, differentiating as Th1 memory cells and remaining refractory to Treg inhibition, and finally preventing the conversion of effector T cells into Treg cells.

The published data are a genuine combination of successes and failures, precluding the drawing of firm conclusions regarding the precise role of Treg. These cells are probably a key element in fighting tumors, and their study is therefore increasingly important.

In order to understand these immunosuppressive phenomena in our model, we designed a therapeutic vaccination following the same pattern as that previously assayed, but with eight million cells/dose. With this dose, the aim was to determine whether our lack of success was due to a lack of antigen/cytokine quantity, or whether the contrary were true; if this high dose proved toxic, Treg were truly the cells involved in inhibition of the response, using confocal microscopy. According to the results obtained, the latter possibility seemed to be correct: the tumor inhibition results were lower with 8 million cells than with those obtained with two million. With blood samples, we proceeded to evaluate Treg cells using confocal microscopy. The results allowed us to draw two conclusions: (a) apparently, classic Treg cells (CD4^+^CD25^+^Foxp3^+^) do not vary their percentages in our model; and (b) cells visualized in the experiment with CD4^−^CD25^+^Foxp3^+^ staining did appear at differentiable rates between different groups, and particularly showed greater proportions in the B16-GM-CSF, B16-B7.2 and B16-GM + B7.2 groups.

These CD4^−^CD25^+^Foxp3^+^ cells may belong to the subset of regulatory T cells CD8 [43], or to other types of regulatory populations such as myeloid suppressor cells, as proposed by various groups, especially that of Ivan Borrello [45,63,64,65,66]. In order to correctly define this population, it would be necessary to conduct new experiments focusing on other types of marking, to help us to clearly identify this population, which seems to be that responsible for the suppressor phenomena in our model. 

## 3. Experimental Section

### 3.1. Plasmids

All the p2F plasmids employed were derived from the pVITRO2 base plasmid (Invivogen, Toulouse, France), employing the empty plasmid (p2F-Ø), or *mGM-CSF* and *mB7.2* genes. pVITRO2 allows the coexpression of two genes and contains two human ferritin composite promoters, FerH (heavy chain) and FerL (light chain), combined to the SV40 and CMV enhancers, respectively, and the resistance to hygromycin gene. Another plasmid was also employed, pMok_mGM-CSF (Mologen, Germany), in order to have another construction expressing *mGM-CSF*, that had previously been successfully employed in our laboratory. This pMok_mGM-CSF plasmid contains the kanamycin resistance gene and the murine GM-CSF gene, controlled by the CMV promoter.

All plasmids were amplified in *Escherichia coli* DH5α, in selective LB broth (Pronadisa, Madrid, Spain), and extracted with the Qiagen Giga Endo-free kit (Izasa S.A., Barcelona, Spain), quantified by spectrophotometry and tested via electrophoresis to confirm their integrity and purity.

### 3.2. Cells and Transfection Procedure

B16 murine melanoma cells were used in all of the experiments. These cells are syngeneic with the animals used for vaccination, *i.e.*, C57BL/6 mice (Harlan, Gannat, France).

B16 cells are adherent cells that were grown in flasks with DMEM (Dulbecco’s modified Eagle’s medium) (Sigma, Madrid, Spain), supplemented with 10% heat inactivated fetal bovine serum (FBS) (Biomedia, Boussens, France), penicillin (100 U/mL) and streptomycin (100 μg/mL). The cells were cultured in a humidified incubator with 5% CO_2_ at 37 °C, and detached from the flasks with Trypsin-EDTA.

The B16 cells employed for the vaccines were transfected by means of a chemical procedure based on PEI 25 kDa (polyethyleneimine, Sigma, Madrid, Spain) polyplexes (DNA:PEI, 1:1.41) with 20 μg/mL of plasmids, as previously described [25,26,67]. The transfection percentage with this method lies between 20% and 40% of total cells, as observed using the reporter EGFP gene (data not shown). Cells were transfected when more than 80% confluence was reached in their flasks. After transfection, we waited for 72 h to irradiate the tumor cells with 150 Gy, and then froze them in DMSO 5% in FBS, with storage at −80 °C or −150 °C until use.

### 3.3. ELISA of m-GMCSF

GM-CSF production in the transfected B16 cells was determined by enzyme linked immunosorbent assay (ELISA), performed on supernatant samples of the culture media taken 72 h posttransfection and prior to cell detachment and irradiation, having changed the media every 24 h. The BD OptEIA ELISA kit for m-GMCSF (Pharmingen, BD Biosciences, Madrid, Spain) was used. A time-point of 72 h was chosen on the basis of prior experimental results, assessed to study cytokine production over time, using the referred transfection conditions [25,26,27,67], in order to guarantee adequate GM-CSF production according to the literature, *i.e.*, >35 ng/10^6^ cells/24 h [24,39].

### 3.4. Preventive Vaccination Procedure

C57BL/6 mice (8–10 weeks old) kept under standard laboratory conditions were housed 5 mice per cage and all animals were vaccinated. All the experiments were approved by the Biological Research Committee of the University of Valencia (Valencia, Spain). In all cases, mice were vaccinated subcutaneously (right leg) with a single dose per week, in weeks −3, −1 and +1 (days −21, −7 and +7), with respect to tumor injection (day 0) with 105 wild type B16 cells in the left leg. The number of cells employed in each vaccine dose was 2 × 10^5^ cells per mouse or 5 × 10^5^ (as indicated) in 100 μL DMEM. 

In all vaccination experiments, blood samples were taken from all the animals and pooled for the same group at each time-point. The samples were taken on days −22 (before any manipulation of the animals, serving as base level or control in each group), −1 (the day before tumor implantation), and +15 (one week after the third and last dose was administered). Plasma was obtained by centrifugation at 3000 rpm for 5 min., and kept at −20 °C until use.

The treatment groups were: (a) B16-B7.2, 2 × 10^5^ B16 cells transfected with p2F-mB7.2 plasmid; (b) B16-GM-CSF, 2 × 10^5^ B16 cells transfected with p2F-mGMCSF plasmid; (c) B16-pMok_GM-CSF, 2 × 10^5^ B16 cells transfected with pMok_mGM-CSF; (d) B16-GM-CSF + B7.2/200, 2 × 10^5^ B16 cells transfected with p2F_mGM-CSF + B7.2; (e) B16-GM-CSF + B7.2/500, 5 × 10^5^ B16 cells transfected with p2F_mGM-CSF + B7.2; (f) B16-p2FØ, 2 × 10^5^ B16 cells transfected with p2FØ; (g) B16*, 2 × 10^5^ B16 nontransfected cells; and h) control, in which only 100 µL DMEM in each vaccination dose were injected. 

### 3.5. Therapeutic Vaccination Procedure

In this set of experiments, with the same basis as in Preventive models, the design was as follows, only changing the cell doses, days of vaccine administration and days of blood retrieval: on day 0, tumor was implanted in the left leg with a dose of 20,000 B16 cells. Vaccination doses were administered subcutaneously on the right leg on days 3, 10 and 17 with respect to tumor injection. The blood samples were taken on days −1, 8, 15 and 22.

Two different doses of B16 transfected cells were evaluated as treatment: 5 × 10^5^ or 2 × 10^6^ cells per dose, per mouse. The vaccination groups were: (a) control (DMEM only); (b) B16-p2FØ, 2 × 10^6^ cells; (c) B16-GM-CSF, 5 × 10^5^ cells; (d) B16-GM-CSF, 2 × 10^6^ cells; (e) B16-GM-CSF + B7.2, 5 × 10^5^ cells; and (f) B16-GM-CSF + B7.2, 2 × 10^6^ cells.

### 3.6. Tumor Growth Measurement and Survival

Tumor growth in mice was monitored visually and measured with a caliper in two dimensions: a (long diameter) and B (short diameter). Tumor volume was calculated with the formula: *V* = (A × B2)/2, and expressed in mm^3^. Animals were collected at date of death to construct the survival curves.

### 3.7. Specific Anti-TMP IgG ELISA

Measurement of IgG and IgG1 and IgG2a subclass antibodies against TMP (Tumor Membrane Proteins) was performed in serum samples by specific ELISA, as previously described [27,38]. TMP is an extract of the hydrophilic membrane proteins of the irradiated B16 cells; thus, with this ELISA we tested the specific response to our vaccine treatment, discarding any other nonspecific immune responses [27,38,68].

Briefly, plates were coated by overnight incubation of TMP at 0.8 μg/mL in carbonate buffer, pH 9.6. The next day, plates were neutralized with 1% BSA solution before the addition of serum samples. For analysis, sera were diluted in dilution buffer (PBS-BSA 1%, Tween 20 0.1%) at 1:1000 for total IgG and IgG1 subclass, and at 1:100 for IgG2a. Bound antibodies were detected with goat antisera to total IgG (Biocheck, Foster City, USA) at 1:10000 or mouse IgG subclasses at 1:1000 (Sigma, Mouse monoclonal isotyping reagents, Madrid, Spain), followed by 1:5000 dilution of biotinylated rabbit antiserum to goat IgG (Sigma, Madrid, Spain) and streptavidin coupled to horseradish peroxidase (Sigma, Madrid, Spain). Plates were developed with a mixture of orthophenylenediamine (OPD, Sigma, Madrid, Spain) and hydrogen peroxide (Fluka-Sigma, Madrid, Spain), and read at 492 nm. All samples were assayed in duplicate, allowing for estimation of the mean OD value and standard deviation.

### 3.8. Characterization of Regulatory T Cells by Confocal Microscopy

In each group the lymphocytes were stained with Mouse T regulatory cell staining kit #2 (eBioscience), following the manufacturer’s instructions. Once completed, cells were moved to LabTek chambers for confocal microscopy. There, 3 µL Hoechst 1mg/mL (Invitrogen) were added to the wells, incubating three minutes in darkness, to allow the total counting of cells/well. Then the cells were scanned with a confocal microscope (Leica TCS-SP2) with laser argon and helium-neon, attached to an inverted microscope (Leica DM1R13).

Finally, the number of cells per field of each of the marks were counted, randomly taking between 5 and 10 different cells from each sample field, and preparing images overlap using the LCS Lite (Leica) program.

### 3.9. Statistical Analysis

Statistical comparison of the tumor growth inhibition results in the different treatment groups was based on two-way analysis of variance (ANOVA) with Bonferroni *post hoc* testing (95% confidence interval, 95% CI), expressing statistically significant differences as indicated in the figures. The same test was applied to the results of the ELISA assays.

Significance in relation to survival was analyzed using the Kaplan-Meier survival curves and the nonparametric log-rank test.

All the tests and graphs were performed with the Graph Pad Prism 4^®^ package.

## 4. Conclusions

Our preventive vaccine model with genetically modified cells reached maximum success: 100% survival in animals vaccinated with nonselected cells transfected to produce GM-CSF. This vaccine, with a reduced cell number (200,000), generated a potent immune memory that allowed the animals to survive a second tumor rechallenge one year later, with only one supplementary vaccine dose. 

Employing bicistronic plasmids, B16 cells expressed not only GM-CSF but also the costimulatory molecule B7.2. This coexpression has been reported to allow the selection and purification of truly transfected cells, which could be a very useful tool for reducing the number of cells required for successful vaccination. In this work, we show that, still in the preventive vaccine model, the results with cells producing GM-CSF and B7.2 were remarkable (80% survival, three months after tumor implantation, 60% for the whole experiment), even with half the amount of GM-CSF produced.

In the therapeutic vaccine model, tumor growth inhibitions of approximately 50% were reached, employing nonselected GM-CSF and GM-CSF + B7.2 producing cells. The limit in the therapeutic efficacy was probably due to immunosuppressor mechanisms triggered by tumor implantation. In our case, the classical Treg cells seem not to be as relevant as in other described settings. However, in our experiments, another type of cells seemed to be implicated in the tolerance phenomena: the CD4^−^CD25^+^Foxp3^+^, but their exact role must be further studied. 
